# Compelled and constrained migration: restrictions to migration agency in the Marshall Islands

**DOI:** 10.3389/fclim.2023.1212780

**Published:** 2023-08-04

**Authors:** Hugh B. Roland

**Affiliations:** Department of Epidemiology, School of Public Health, University of Alabama at Birmingham, Birmingham, AL, United States

**Keywords:** environmental migration, climate change, agency-structure, geographic isolation, adaptation, Marshall Islands

## Abstract

Migration as adaptation implies agency, yet environmental and non-environmental factors and their interactions may limit the availability of adaptation options, including migration. This study investigates migration agency in the Marshall Islands, particularly the role of geographic isolation and climate change. Interviews with internal migrants living in Majuro and members of government and civil society reveal how social, economic, cultural, and environmental factors shape migration contexts. Results suggest that geographic isolation-related factors may increase likelihoods of simultaneously more compelled and more constrained moves, particularly as climate change impacts increase. Climate change-related impacts on resource-dependent livelihoods may compel migration in search of new economic opportunities. However, worsening environmental conditions may also exacerbate cost-related migration constraints by reducing the resources available to support migration.

## Introduction

Popular media frequently depict the Republic of the Marshall Islands (RMI), a low-lying atoll nation, as “sinking” due to sea level rise, with the implication that the nation’s inhabitants will have to relocate as climate change refugees.^1^ The climate science on sea level rise and other climate change impacts may seem to justify these fears: Sea-level rise is projected to gradually cover much of the country’s land area (Pachauri and Meyer, 2014; Owen et al., 2016), recent estimates of the economic toll of sea level rise in island nations reveal greater impacts than originally estimated (Martyr-Koller et al., 2021), and research warns that, without radical adaptation, inundations and contamination of freshwater lenses threaten atoll nations’ habitability (Barnett and Adger, 2003; Storlazzi et al., 2018). However, how climate change-related impacts translate to human impacts is less clear, and mass forced migration cannot be assumed to be inevitable (Barnett, 2017; van der Ploeg et al., 2020).

Developments in climate mobilities research have critiqued narratives of climate change-induced mass migration (Boas et al., 2022). Citing millennia of Indigenous adaptation and resilience in the Pacific (Steiner, 2015; Teaiwa, 2018), recent research stresses that habitability is shaped by local knowledge and that the effects of climate change on atoll habitability are contestable (Farbotko and Campbell, 2022). In the face of adverse climate impacts, Indigenous people of the Pacific increasingly prefer to stay on their lands for cultural and spiritual reasons rather than relocate (Farbotko, 2018, 2022). In Tuvalu, grassroots anti-displacement mobilities and reemplacement activities challenge dominant climate mobility narratives and reaffirm Indigenous rights (Farbotko, 2022), and, in the RMI, government and civil society strongly oppose the donor-supported relocation storyline (Bordner et al., 2020).

Research on environmental migration in the Pacific demonstrates variation in the extent to which climate change influences moves, ranging from infrequent references to environmental factors and criticism of climate change-driven migration narratives in Tuvalu (Mortreux and Barnett, 2009) to internal relocation within atolls as an adaptation strategy in French Polynesia (Duvat et al., 2022) to climate change concerns influencing future out-migration in Kiribati (Allgood and McNamara, 2017). Nevertheless, a recent systematic review refutes assumptions that migration due to climate change is already happening across atoll nations (Mortreux et al., 2023). In the RMI, research identifies education, healthcare, and economic and social factors as dominant migration drivers (van der Geest et al., 2020), with economic hardships that frustrate abilities to meet basic needs and new demands of changing lifestyles of particular concern (Rudiak-Gould, 2013, p. 148).

While climate change does not currently seem to be a major direct driver in the RMI, climate change impacts may influence migration via economic and social factors, such as shocks’ effects on subsistence livelihoods and food security (Oakes, 2019). For example, outer island residents, especially, face high and increasing risks from dwindling sea life, declining rainfall, increasing erosion, and higher heat extremes that damage crops, which may influence adaptation and migration abilities and decisions (Rudiak-Gould, 2013, p. 57). The influence of non-environmental drivers reflects the interactive role of economic, political, demographic, and social drivers with environmental changes and highly contextual migration patterns, including existing migration corridors and non-climate anthropogenic drivers of risk, that make it difficult to reduce migration to a single influence (Black et al., 2011; Baldwin et al., 2019; Magnan et al., 2022). Indeed, a growing number of studies find that relationships between climate change and migration are more often indirect, small, and shaped by local contexts (de Sherbinin, 2020; Hoffmann et al., 2020; Boas et al., 2022).

A reductionist emphasis on climate change impacts driving migration raises questions regarding both the accessibility and necessity of migration as an adaptation strategy. While many mobility options were available to and practiced by Indigenous peoples in the Pacific, these options may be increasingly limited by degraded infrastructure, expensive travel costs, and political barriers (Boas et al., 2022). Climate change-related impacts on economic or social stability may reduce migration capacities (Warner et al., 2010; Zickgraf and Perrin, 2016), and damage to infrastructure necessary to support migration may block immediate moves, although infrastructure damage or disinvestment can operate as a migration push if poor infrastructure decreases accessibility and habitability (Cook and Butz, 2015; Hock et al., 2019; Olsen et al., 2021; Blondin, 2022). Socioeconomic and political contexts and colonial legacies also restrict adaptive capacities, increasingly compelling moves in some circumstances and undermining moving abilities in others (McLaughlin and Dietz, 2008; Bettini and Gioli, 2016; Bordner et al., 2020; Boas et al., 2022). For example, research in neighboring Tuvalu and Kiribati found that people were interested in migrating but unable to move, largely because they lacked the financial means (Milan et al., 2016; Oakes et al., 2016).

Recognizing that migration is often less preferred than in-place adaptation, this study investigates agency associated with migration in the RMI—how migration may be compelled or constrained—by examining common migration drivers and the roles of climate change and geographic isolation. Under what circumstances is migration a household or individual choice vs. forced or blocked? If few adaptation options are available, to what extent is any option a choice? Agency refers to the capacity of individuals and groups to act freely in culturally informed ways (Lazrus, 2012). Other definitions use different terminology to reflect similar concepts—the ability to act on one’s values (Ibrahim and Alkire, 2007). Structure refers to sociopolitical, cultural, and economic systems that limit and inform choices and opportunities (Rossi, 1993).

Migration scholars have proposed several framings to understand the role and extent of choice in migration (McLeman and Hunter, 2010; Baldwin, 2014). Aspirations and abilities models, for example, aim to explain why some people move while others remain in place by assessing migration-related perceptions and desires and factors that limit abilities to act on aspirations (Carling, 2002; De Haas, 2021; De Sherbinin et al., 2022). Central to these models is reasoning that migration occurs along a voluntary-to-forced continuum, where more voluntary migration requires that origin conditions do not threaten severe conditions like health, starvation, or extreme poverty and that migrants are neither coerced to move nor without a bearable exit option (Hugo, 1996; Bakewell, 2010; Ottonelli and Torresi, 2013; Bartram, 2015). Despite this attention to agency in the broader migration literature, reviews of environmental migration research note limited scrutiny in environmental migrations (Obokata et al., 2014; Hunter et al., 2015). Environmental migration research considers agency in describing political and economic structures and inequalities that limit migration options and abilities (Alscher, 2011; Wrathall et al., 2014), including in the Pacific (Birk and Rasmussen, 2014), but agency is rarely a focus. However, growing attention in environmental migration research to the social, cultural, economic, and political factors that set contexts of environmental adaptation supports evaluation of migration agency related to these factors (Obokata et al., 2014; De Sherbinin et al., 2022). Agency also varies widely within communities (Sheller, 2018). Diversity in movement is shaped by individual and family capabilities and aspirations (De Haas, 2021), including resources necessary to relocate (Boas et al., 2022) and vulnerabilities that can increase risks of involuntary displacement (Adger et al., 2018).

Migration agency in the RMI is important to examine as the islands’ geographic isolation and the distance between outer islands and the main island, Majuro, and between Majuro and the primary international migration destination, the United States, may generate exceptional vulnerability and agency-related conditions. The Pacific Islands have resilient, prosperous, and interconnected precolonial histories, with geographic isolation and vulnerability a product of how colonialism and capitalism refashion space (Hau’Ofa, 1995; Gaillard, 2007; Reenberg et al., 2008; Maldonado et al., 2013; Mitchell-Eaton, 2021). Related to the RMI’s colonial history, limited economic opportunities, funds to invest in local adaptation, and access to goods and services may frustrate risk diversification and create circumstances where migration is the only feasible option (Kelman, 2007, 2020; Shultz et al., 2016; Doogan et al., 2018; Kim and Bui, 2019). These circumstances may restrict adaptive capacities and support the environmental scarcity thesis, where poor environmental conditions prompt out-migration in search of more secure livelihoods (see also Gray, 2009 for similar logic though different terminology; van der Geest, 2011, pp. 128–129; Hunter et al., 2015). Concurrently, however, high transportation costs may block moves. Difficult and costly moves and long migration distances constrain migration capacities (Henry et al., 2003; Gray, 2010; Massey et al., 2010; Nawrotzki et al., 2013; Birk and Rasmussen, 2014), and worsening conditions that affect livelihoods necessary to support challenging moves may lead to less long-distance migration, particularly for older and lower-income residents (Findley, 1994; Warner and Laczko, 2008; Alscher, 2011; Findlay, 2011; Roland and Curtis, 2020). These circumstances reflect the environmental capital thesis, where declining environmental conditions block out-migration by limiting access to resources needed to support migration (Gray, 2009; van der Geest, 2011, p. 128–129; Hunter et al., 2015). Noting the importance of scrutinizing contexts within which adaptation and migration decisions are made (Hoffmann et al., 2020), this study pays particular attention to how factors related to islands’ geographic isolation may affect agency, including the possibility of opposing forces, where dynamics both compel and constrain migration.

Evaluating migration agency in a context that popular media frequently refers to as “ground zero” for climate change-related migration is important as debates around agency-related migration terminologies have wide-reaching policy and human consequences (Erdal and Oeppen, 2018). What conditions are considered sufficiently severe to classify migration as forced and what constitutes a bearable migration option are subject to normative beliefs and often defined by actors in the Global North. Migrants’ perspectives on the circumstances of their own migrations seldom factor into classifications. Already, the political stakes surrounding classifications of voluntary vs. forced shape debates about definitions (Ottonelli and Torresi, 2013; Erdal and Oeppen, 2018). Environmental justice advocates may favor the term “climate change refugee” for suggesting a climate debt industrialized nations owe to those migrating, and, following similar reasoning, policymakers in the Global North may avoid the term (White, 2011; Felli, 2013). While Pacific Island leaders reject the refugee label for its disempowering connotations, some researchers argue that avoiding refugee classifications fails to capture the degree to which the environment affects adaptive capacities, particularly in cases where migration is one of few adaptation options (Farbotko and Lazrus, 2012; Lazrus, 2012). Acknowledging patterns of mixed migration, where people move along the same routes for different reasons, and the implications of migration classifications for siloed policy responses, scholars have called for a conceptual reconsideration of voluntary and forced distinctions (Landau and Segatti, 2009).

Recognizing a need to reexamine migration categorizing and classification (e.g., Renaud et al., 2011) and observing calls for research on factors that enable or hamper human mobility (Blondin, 2022) and scrutiny of policies and institutions that might support or obstruct local agency (Mortreux et al., 2023), this study uses interviews with a sample of internal migrants living in Majuro to examine perceptions of migration drivers and migration-related agency. Mobility is core to Marshallese identity (Jarillo and Barnett, 2021), and research on how migrants perceive the drivers that influenced their migration, their ongoing mobility, and their migration experiences, including challenges and barriers, may highlight disparities in adaptive capacities and inform policy to support the accessibility of multiple adaptive responses.

### Marshall Islands migration context

The RMI has a long history of resilience, adaptation, and interconnectedness and mobility (Teaiwa, 2018; Jarillo and Barnett, 2021; Mitchell-Eaton, 2021). The RMI is also the site of the U.S.’ nuclear testing, and the legacy of the 67 nuclear tests, and related colonial histories, have greatly influenced contemporary Marshallese social, cultural, economic, and political contexts. Part of this legacy is an agreement that the RMI has with the U.S.—the Compact of Free Association—that permits Marshallese to live and work visa-free in the U.S. and blurs distinctions between internal and external migration. Recent research estimates that over a third of Marshallese reside outside of the RMI (30,000 in the U.S.), concentrated in Hawaii, Arkansas, and Washington and primarily employed in low-wage service jobs and the poultry industry (Yamada et al., 2017; van der Geest et al., 2019).

While dated, the most recent 2011 census contextualizes migration in the RMI (Republic of the Marshall Islands 2011 Census Report, 2012). In 2011, 52.3% of the country’s 53,158 residents resided on Majuro, with urbanization increasing: 73.8% of the population lived in urban areas (Majuro and Kwajalein atolls) compared to 65.2% in 1999. For many from outer islands, Majuro is a temporary stop where migrants can work cash jobs and save for the expensive flight to the U.S. In the 10 years preceding the 2011 census, the population declined on almost all outer islands. Emigration and, to a lesser extent, a decline in fertility has driven declining population growth since 1988. The population’s 2011 dependency ratio was 72.3, meaning that each 100 people of working age (15–64) in the population support 72.3 children (0–14) and elderly residents (65+). This ratio is declining, which indicates an increasing percentage of the population in working ages and possible related economic and out-migration pressures. Preliminary results from the 2021 census show a 26% drop in the population, overwhelmingly due to out-migration (Johnson, 2021).

## Materials and methods

Data collection was conducted in Majuro from January to March and September to November 2019 with support from two organizations, the Marshall Islands Conservation Society (MICS) and the RMI office of the International Organization for Migration (IOM). These organizations were already involved in related projects and research on climate change adaptation, disaster response, and migration. Staff from these organizations and the RMI Office of the Chief Secretary assisted in developing research questions, identifying appropriate methods, and reviewing protocols.

Primary data collection involved 28 in-depth interviews with adult migrants from outer islands living on Majuro. Interviewing internal migrants of varying social and economic backgrounds and from different origin islands was necessary for comparisons between migration contexts, drivers, and experiences. Participants were sampled from a 2018 IOM internal migrant household survey. Interviews were intended to contextualize IOM survey results, which was a need highlighted by IOM staff and in the survey’s final report (International Organization for Migration, 2018). The survey contacted 1,215 individuals across Majuro. Interview participants were recruited from survey respondents who had moved from an outer island, indicated a willingness to be recontacted, and provided an email address or phone number (n=220, 82 men and 138 women). Prospective participants were grouped by island of origin and randomly recruited from these groups to ensure a mix of migration origins in the sample. Interview participants varied widely by age (age 19–70), gender (6 men, 22 women), occupation, socioeconomic status, place of residence in Majuro, migration origin, and time of migration (arriving in Majuro between 1.5 and 41 years prior). Participants were mostly women as 63% of survey respondents were women. Participant variation was important to compare migration experiences.

The study’s sample has several constraints. The overrepresentation of women may lead to overemphasis of certain drivers and migration experiences. This bias in the sample may be due to greater likelihoods of men migrating long distances and continuing to the U.S. and, thus, more women remaining in Majuro (Ravenstein, 1885; Silvey, 2006). Additionally, no non-migrants and ongoing migrants to the US were interviewed. Inclusion of these populations might offer a more complete picture of migration drivers, experiences, and agency in the RMI. Finally, the sample is more representative of non-circular internal migrants and longer-term internal migrants to Majuro as internal migrants to Majuro often continue to the U.S. within a few months of arriving on Majuro yet the time lag between survey and interviews prevented follow up data collection with these migrants. As the international move is expensive, migrants in the study sample may thus be less financially resourced than migrants who continue to the U.S. shortly after arriving on Majuro (Findley, 1994; Findlay, 2011).

Five interpreters supported participant recruitment and interview conduct. Four interpreters worked with MICS or IOM, which meant that they were familiar with the subject matter. This large number of interpreters was necessary for interview scheduling but also increased the number of key informants informing the research process and allowed for interpreter-participant matching by gender when schedules permitted (Edwards, 1998). Interpreters were trained together to promote consistency and encouraged to identify and ask follow-up questions to minimize translation-related disruptions (Adamson and Donovan, 2002; Williamson et al., 2011). Post-interview, interpreters translated and transcribed recordings.

Data collection also included 12 key informant interviews with elected officials, government staff, and members of civil society, conducted in English without interpreters. Participants included mayors, senators, and leaders of aid organizations and government departments. These interviews provided context on the conditions surrounding migration in the RMI and highlighted wider social implications and possible solutions. Senators and mayors from outlying islands discussed the specific circumstances on the atolls they represented.

Thematic analysis of both sets of interviews was conducted in NVivo. Codes were identified related to participants’ migration influences and experiences and descriptions of vulnerability and resilience on outer islands and Majuro and organized thematically. To support accurate analysis, key points from interviews were discussed with interpreters following interviews and initial codes reviewed at the end of data collection (Nowell et al., 2017). Results include frequent use of direct quotations to reflect participant voice.

## Results

### More compelled migration

Shifting social, economic, and environmental circumstances and varying abilities to meet individual and community needs on outer islands may lead to more compelled moves. Internal migrants reported access to healthcare, education, and economic opportunities as the most common migration drivers and described how limited access to goods and services on outer islands necessitated many of these moves. Within migration decision-making processes, participants described agency varying across demographic and social characteristics, including age, sex, socioeconomic status, and familial ties. For example, several women commented that migration decisions were made by their husband and that they would not have moved had it been their decision.

#### Moves to access healthcare and education

Limited access to services on outer islands drives migration for healthcare and education. Linking geographic isolation-related healthcare and education barriers to compelled migrations, one participant observed:

People aren’t forced to move here (to Majuro), but they have to move here to find a better job, to go to school, to get healthcare services. There’s a school in Namu, but only elementary. There are clinics there, but they don’t have enough supplies or services.

(Middle-aged woman from Namu)

Participants described education-related moves as common and necessary since most outer islands do not have schools beyond primary levels. Accordingly, participants discussed education-related migration decision making in terms of determining duration and destination rather than whether to move. Similarly, participants described limited access to medical care on outer islands compelling moves for both those seeking healthcare and caregivers. Recounting one such move, one participant noted that her parents did not want to leave the outer islands but made the move to care for her grandmother: “My grandmother was sick, so we came here. My grandma was already living here, and we moved here to take care of her… Neither of my parents wanted to move (to Majuro), but they came to take care of my grandmother” (Middle-aged woman from Ebon). Describing a similar situation, another participant observed greater agency in return migration following a medical move:

When it comes down to a patient with no further things they can do for them on the outer island, they have no choice but to move here because there is only one health assistant on every outer island and they can’t cure a lot of patients…At the time my mum was sick, it was both I had to come and I wanted to come. And it was my choice to stay here. I could have gone back but because everyone had already moved here, we just stayed.

(Middle-aged woman from Ailinglaplap)

Returning to outer islands following medical treatment is only an option, however, when care does not require regular checkups or medicines unavailable on outer islands. Social and economic factors may also encourage migrants to stay in Majuro and lead medical moves intended as temporary to become permanent. One participant shared such a scenario, explaining how having multiple family members come to Majuro to support a medical procedure encouraged permanent resettlement since the social network was now in Majuro:

I moved here to give birth. My parents moved with me. They came to be with me for the birth. They didn’t plan to stay. My dad had a job on Jaluit. He took leave to come here, but two weeks after he got here, he was offered a job and he took it. We didn’t stay because of the job but because everyone was here already.

(Older woman from Jaluit)

These accounts suggest that circumstances surrounding education and medical care on outer islands, where accessing many services is impossible without moving, create situations where migration to meet these needs may feel more compelled.

#### Moves to access economic opportunities and provide social support

Limited economic opportunities on outer islands may compel migration via social factors. Social support is common throughout the RMI and necessary to support climate change adaptation. However, support often requires access to cash, and for those living on outer islands with few economic opportunities, migration may be necessary to find cash work. Many individuals thus move to Majuro or the U.S. to support family on outer islands. One participant described this dynamic, noting especially the effect of high and rising costs of imported goods on outer islands:

There are a lot of changes in the prices, and I feel that might be one of the reasons why people move from the outer islands to here, to be able to make more money to buy food. They come here to be able to support those who are back home on the outer island.

(Middle-aged woman from Ebon)

Conditions in migration destinations may drive outer island out-migration for similar social support reasons. Several participants discussed moves to help family in Majuro and the U.S. For example, one participant described moving to Majuro to support her adult son despite wanting to stay on the outer islands: “It was sort of hard for us because we didn’t want to leave our island, but we had to because we had to support our son” (Middle-aged woman from Maloelap). Other participants reported migrating to take care of relatives’ children so that parents had more time for cash work: “The reason I came to Majuro was because my older sisters wanted me to look after their kids while they went out to work…This was the only reason” (Older woman from Maloelap). Moving to care for relatives’ children is especially common in U.S.-bound migration as migrants to the U.S. often work long hours in low wage jobs and have little time or money for childcare. Describing this situation, one participant linked recent migrants’ financial conditions to further migration within the social network: “Sometimes the Marshallese in the States like to get some more people from over here to go over there so that they can babysit for them or also work to help pay the bills” (Young man from Likiep). Participants’ observations of few alternative options to support family members and reports of reluctant moves suggest that some of these migrations feel obligatory.

#### Moves to diversify resource-dependent livelihoods

Environmental changes may increasingly compel migration in search of new economic opportunities by reducing the viability of resource-dependent livelihoods on outer islands. While downplaying direct environmental influences on migration, participants stressed effects of environmental shocks and shifts on livelihoods, which in turn may influence migration. Several participants reported moves due to saltwater intrusion affecting subsistence livelihoods. For example, one participant noted: “When the tide rises, the saltwater damages some of the areas and we are no longer able to plant our food in those areas. I have a relative who moved because of this” (Middle-aged woman from Ebon). Participants also emphasized environmental impacts on copra production, which is the primary cash livelihood on outer islands. Referencing the environmental scarcity thesis, where environmental degradation prompts out-migration in search of more secure livelihoods, participants warned that environmental changes affecting copra would increasingly compel moves. One participant projected such migration pressures:

I think that the less and less coconuts they (outer island residents) have available to harvest and make copra would cause migration. Because that is their main source of income in the outer islands. If that happens (copra production declines), they would not be able to make money. I think that’s the main problem I see that could cause them to move.

(Young man from Ailinglaplap)

Migrants also described neoclassical migration dynamics when discussing copra production. For example, several participants discussed moving in search of higher wages and easier work:

In Wotje, there are a lot of people who are tired of working with copra and they want to work a different job so they come here to get different jobs.

(Middle-aged woman from Wotje)

That is the main reason why I moved. I did not want my children to end up like me—making copra and having low paying jobs. I wanted them to have a better life. I have seen how hard it is to make copra.

(Middle-aged woman from Milli)

As environmental conditions decline and livelihoods become more strenuous, neoclassical dynamics may become intertwined with environmental pressures and increasingly overlap with more compelled environmental scarcity dynamics. These dynamics risk conflicting with migration-constraining dynamics associated with the environmental capital thesis, where declining conditions reduce resources available to support costly moves.

### More constrained migration

While connectivity within and from the RMI has increased in recent years, mobility depends on being able to afford expensive travel. Costly travel may be particularly inaccessible to cash-poor residents, outer island residents with limited cash-earning opportunities, and residents without strong familial and social networks to support a move. For internal migrants living in Majuro, wide socioeconomic disparities also create contrasting ongoing and return migration capacities and experiences. Many cash-poor participants described travel costs limiting moving abilities, particularly return, circular, and on-migration.

#### Moves blocked by travel logistics and costs

Logistical challenges are common in migration within and from the RMI and may discourage or block moves. Inter-island transit remains difficult despite more transportation links. Flights are often full and too expensive for most, and, consequently, internal migration depends on infrequent and unpredictable boat travel. Boat schedules change often, travel is weather-dependent, and trips typically take multiple days. Travel logistics are particularly difficult for populations farther from Majuro as boats load passengers and goods at intermediary stops and may not have space for additional passengers. Describing boat travel from one of the outer islands farther from Majuro, one participant noted: “Aur is usually the last destination for the boats when they go around the outer islands, and it’s usually pretty full for more people to get on” (Young man from Aur). For many migrants, difficult travel logistics discourage and even prevent moves. Weather may also upset travel plans. One participant recounted weather blocking a move: “At the time that my dad was going to go, the ocean was rough and it was not a good time to travel, so we stayed” (Middle-aged woman from Jaluit). The deterrent effect of difficult travel is particularly common in circular and return-migration. Describing how travel difficulties discouraged him from visiting family on outer islands, one participant shared: “(When we travel to outer islands to visit), we usually get stuck there due to transportation schedules changing” (Young man from Wotje).

Cash-poor participants especially described difficult and expensive moves limiting migration agency. Participants from different socioeconomic backgrounds shared contrasting perceptions of costs and accessibility. A wealthier participant noted cost-related inaccessibility affecting a few people:

On a boat it’s about $20 (USD) to come but on plane it’s $150 (USD). It seems like it’s a low enough price that a lot of people are able to move, but there are a few people who don’t move because they don’t have the money to.

(Middle-aged woman from Wotje)

In contrast, cash-poor participants described high travel costs as more widely preventative. A less affluent participant recounted constraints associated with spending 70 USD on boat travel from an island farther from Majuro. Another recounted financial hardship blocking an international move to access healthcare: “For my sister (in Majuro), her husband doesn’t work, just her children, and so it’s hard for them because they want to move to places with better doctors (e.g., the U.S.), but they don’t have enough money to do that” (Older woman from Maloelap). Participant responses highlighted socioeconomic disparities in accessibility and ease of travel. For example, one participant commented that more affluent individuals and families are far more able to travel and travel comfortably: “For those with financial stability, it is easy (to move), but for those with financial strains like me and my family, it is very difficult to move around” (Young woman from Ailinglaplap). Expanded transportation networks have increased travel abilities for wealthier residents, but, as less affluent participants stressed, high costs have reduced accessibility for others.

Travel costs that require prospective migrants to plan and save for trips may severely reduce rapid moving capacities. Cash-poor participants reported that they and their families spent months working to save for moves, and saving for moves may be complicated by few cash-earning opportunities on outer islands: “I did copra making to save up for my trip here… It took a long time” (Young woman from Ailinglaplap). Prospective migrants without strong networks in migration destinations face even larger cost barriers. As many migrants rely on family to pay for travel, migration capacities, and rapid migration capacities especially, may depend on prospective migrants’ networks in migration destinations. However, even with social support, saving for travel costs can take time: “It took a lot of time to save up for a ticket. Eventually our relative paid for my ticket” (Older woman from Ailinglaplap). Widespread practices of relying on months of personal and familial network savings for moves has critical disaster response implications. Observing the extensive planning typically needed for moves, several participants doubted that they would have the resources necessary to move should environmental conditions demand immediate migration. These concerns suggest dynamics that reflect the environmental capital thesis, where declining conditions that affect natural resource-dependent livelihoods and prospective migrants’ cash access make moves less accessible.

#### Return migration and visiting travel especially constrained

The high cost of travel especially limits temporary and circular migration and return migration and visits for cash-poor migrants. One participant recounted how high costs discouraged return trips despite her familial connections:

I haven’t been back since I came here. I miss friends and family there, but we just haven’t visited since we moved in 1986. There aren’t any plans to go back…That’s (the high cost of travel) one of the reasons why I don’t want to visit (outer islands). I don’t want to pay all that money to go and come back.

(Older woman from Jaluit)

This experience contrasts with more affluent migrants’ more frequent travel. One wealthier participant described flying to her outer island often, visiting three times with her children over the previous summer and sometimes visiting for the day to see her parents and swim. Prohibitively high travel costs also limit return trips for migrants in the U.S. The high cost of international travel (a one-way flight to the U.S. in 2019 cost over 1,000 USD) frequently discourages or delays initial migration, but this move is often eventually accessible as prospective migrants save for long periods, rely on family members for support, and have expectations of higher wages in the U.S. High travel costs, however, frequently prevent return migration and return visits. Several participants described this constraint:

It’s really expensive to come back (to the RMI), and so they (those who move to the U.S.) end up staying there forever.

(Middle-aged woman from Likiep)

If we did migrate (to the U.S.), we wouldn’t be able to come back because it’s impossible to afford plane tickets.

(Older woman from Aur)

High travel costs are more limiting for cash-poor migrants but also affect wealthier migrants’ return migration. An affluent participant who had lived in Hawaii for several years explained that she and her siblings only returned to the RMI because her employer paid for their travel. Recounting her migration history, she also noted common experiences of family members moving together:

I had always considered moving back (from Hawaii), but I had so many siblings with me that tickets back home meant a lot (of money). I was in a good place where I had a really nice job, and my siblings were there, we were ok. So, I said I’ll go back once everyone’s finished their school here. But when ***** called and gave me an offer they said they’d pay for my siblings (to come back too) because I said I can’t move without them.

(Middle-aged woman from Likiep)

For most migrants, but cash-poor migrants especially, high transportation costs and difficult logistics reduce migration capacities, particularly return migration and visits. Participants stressed disparities in these constraints and noted possible consequences, including difficulties maintaining ties to outer islands and risks of being unable to afford return trips and becoming trapped in destinations.

### Policymakers support access to multiple adaptation strategies

Emphasizing a need to support multiple adaptation strategies, RMI policymakers shared wide-ranging projects and proposed projects to increase strategies’ accessibility. Echoing findings from recent research (Bordner et al., 2020; Campbell and Bedford, 2022), policymakers favored in-place adaptation and approaches that reduced out-migration pushes, such as economic development. However, policymakers recognized that migration pressures and residents’ varying needs and wishes also required facilitating migration. Examples of migration support include paying for the internal migration of students accepted at the College of the Marshall Islands and organizing predeparture orientations for U.S.-bound migrants. Other strategies such as investments in education and transportation infrastructure support both in-place adaptation and migration. Despite these initiatives, policymakers stressed severely limited capacities due to small budgets and insufficient international support. As in other recent research interviewing RMI policymakers (Bordner et al., 2020), government representatives tied disinvestment that has constrained national adaptive capacities to colonial legacies and aid systems that reinforce dependency and deny local sovereignty and knowledge.

## Discussion

This study examines migration agency in a sample of internal migrants in the RMI, evaluating perceived migration drivers and roles of geographic isolation and climate change. Interviews with migrants living on Majuro and members of government and civil society reveal how social, economic, cultural, and environmental factors shape migration contexts and agency. Building on studies that consider effects of political and economic structures and inequalities on moving abilities (Alscher, 2011; Wrathall et al., 2014) and weigh the influence of geographic isolation-related factors on adaptive capacities (Kelman, 2007; Nawrotzki et al., 2013; Birk and Rasmussen, 2014; Kim and Bui, 2019; Roland and Curtis, 2020), this study reports on participants’ perceived barriers to migration agency. While findings are specific to the RMI, findings contribute to limited agency-focused environmental migration research and may have relevance for other geographically isolated contexts (Obokata et al., 2014; Hunter et al., 2015).

Results suggest that geographic isolation-related factors may increase likelihoods of both more compelled and more constrained migration. Climate change impacts may exacerbate these opposing forces. For example, environment-related impacts on resource-dependent livelihoods may compel migration in search of new economic opportunities (the environmental scarcity thesis). However, despite improved mobility in the last two decades, largely due to rising incomes to support high transportation costs (Jarillo and Barnett, 2021), expensive travel continues to constrain migration for cash-poor residents, particularly return migration and visits. Worsening environmental conditions may exacerbate cost-related migration constraints by reducing the resources available to support migration, for example, if prospective migrants can no longer save for moves by producing copra (the environmental capital thesis). Inaccessible return migration raises the stakes of migration. Less economically successful migrants may want to return yet may be unable to afford travel costs. Cash-poor migrants less able to travel may also struggle to maintain social and cultural ties to origins. Regular return visits may allow more affluent migrants to maintain communal ties on outer islands, supporting an enduring sense of connection and encouraging further trips. However, cash-poor migrants may struggle to sustain similar ties.

Concurrently more compelled and more constrained migration and scenarios where both the environmental scarcity and capital theses apply may exacerbate vulnerability. Changes to the demographic structures of outer islands and Majuro resulting from out-migration may further complicate migration drivers and compelling and constraining dynamics. For example, as many working-age residents leave outer islands, those remaining in-place—largely older residents and children—may struggle to maintain resource-dependent livelihoods made increasingly strenuous from environmental changes. This demographic shift raises questions about the long-term viability of economic migration undertaken to support the in-place adaptation of family members remaining on outer islands (e.g., Farbotko et al., 2022).

This research, highlighting perceived barriers to migration agency and new risks from climate change, has policy implications for enhancing accessibility of adaptation options and reinforces policymakers’ calls for funding necessary to implement the diverse strategies outlined in the RMI’s National Adaptation Plan to strengthen adaptive capacities. Future research might examine the vulnerability implications of simultaneously increasing out-migration pressures and migration inaccessibility, effects of shifting demographic structures, heterogeneity related to population characteristics like age and gender, and projected impacts as environmental shocks and shifts grow increasingly frequent and extreme. Future research might also include control populations of non-migrants and migrants to the U.S. to expand the comparative analysis of migration contexts, drivers, and outcomes.

## Data Availability

The original contributions presented in the study are included in the article/supplementary material, further inquiries can be directed to the corresponding author.

## Footnotes

1 See, for example: Francis, Ellen. November 1, 2021. Marshall Islands pleads with world leaders to stop the 60,000-person nation from drowning. *The Washington Post*, https://www.washingtonpost.com/climate-environment/2021/11/01/marshall-islands-sea-levels-cop26-tina-stege/.
